# Effects of cyclosporin-a on rat skeletal biomechanical properties

**DOI:** 10.1186/1471-2474-12-240

**Published:** 2011-10-24

**Authors:** Yixin Chen, Xin Zheng, Rui Zou, Junfei Wang

**Affiliations:** 1 Department of Orthopedics, Drum Tower Hospital of Nanjing, affiliated to the Medical School of Nanjing University, Zhongshan North Road, No.381, 210008, Nanjing, Jiangsu Province, PR China

## Abstract

**Background:**

Cyclosprin A (CsA) has been widely used clinically to treat the patients who have undergone organ transplantation or acquired autoimmune disease. The purpose of this study is to determine the effects of three different doses of CsA (1.5, 7.5, 15 mg/kg body weight) on the skeletal biomechanical proprieties at different anatomic sites in rats.

**Methods:**

Fifty-six male 3-month-old Wistar rats were divided into five groups. Eight rats were randomly chosen as the basal group, while the others were randomly distributed into four groups of 12 animals each. One group was used as controls and received daily subcutaneous injection of 1 ml of saline solution; another three experimental groups were injected subcutaneously with CsA in a daily dose of 1.5, 7.5, and 15 mg/kg body weight respectively for 60 days. The bone biomechanical proprieties, the bone mineral density, as well as the trabecular bone architecture were measured at different anatomic sites, i.e. the lumbar vertebra, the middle femur shaft, and the proximal femur.

**Results:**

CsA therapy at 7.5 and 1.5 mg/kg can significantly reduce the ultimate force, the ultimate stress and the energy absorption per unit of bone volume of the lumbar vertebra, with no effect on the middle femur. CsA therapy at 7.5 mg/kg can significantly reduce the ultimate force, the ultimate stress and the Young's modulus of the femoral neck, but not CsA at 1.5 mg/kg. Furthermore, CsA therapy at 7.5 and 1.5 mg/kg can significantly reduce the bone mineral density of the lumber vertebra and the proximal femur, but have no effect on the middle femur. CsA therapy at 7.5 and 1.5 mg/kg can also significantly reduce the bone volume fraction of the proximal tibia and the lumber vertebra, but has no effect on the cortical thickness of the middle femoral shaft. In the 15 mg/kg CsA group only one rat survived, and the kidney and liver histology of the survived rat showed extensive tissue necrosis.

**Conclusion:**

Long-term use of CsA can weaken the biomechanical properties and thus increase the fracture rate of the lumbar vertebra and the proximal femur. However, CsA therapy has less effect on the middle femur shaft. The effects of CsA on skeleton are site-specific.

## Background

Cyclosporin A (CsA) has been widely used clinically to prevent organ rejection in post-transplantation and to ameliorate the autoimmune disorders. However, since the patients usually receive a combination of several drugs, the isolated clinical effects of CsA on human skeleton are still unclear. Although it is increasingly believed that CsA can cause bone loss in humans [1,2], it is interestingly found that CsA monotherapy in renal transplantation patients can significantly increase lumbar bone mineral density [3,4]. Yet up to now, a correlation between the daily or accumulative dose of CsA and fracture frequency has not been established [5]. It remains to be found out whether long-term use of CsA will increase the fracture risk in humans.

There are numerous experimental studies evaluating the effects of CsA on the bone metabolism. Some in-vivo studies have demonstrated that CsA in the rats can induce high-turnover bone loss, resulting in an uncoupling of the dynamic bone remodeling cycle with resorption exceeding formation and leading to an ultimate loss of trabecular bone [6-8]. Bone histomorphometry in these studies shows increased osteoclast number, increased parameters of bone formation, and decreased percent trabecular bone volume [6-8]. However, CsA dos not affect the absolute rate of cortical bone resorption at the organ level [9]. The effect of CsA on bone mineral metabolism is dependent on both the dose and the duration of administration [10,11]. Transforming growth factor-beta, alendronate, and raloxifene analog may have potential in modulating the deleterious bone effects of CsA [7,12,13]. The precise mechanisms involved in CsA-induced bone loss are still not well defined. Wada et al suggested that the bone resorption in CsA-treated rats may be activated by plasma parathyroid hormone (PTH) [6]. However, Epstein et al founded that PTH may not be essential for CsA-induced bone loss, and Buchinsky et al suggested that T lymphocytes appeared to be a prerequisite for the development of CsA-induced bone loss [14,15].

Few studies have evaluated the effects of CsA on bone mechanical properties. As Jarvinen et al emphasized, we should not forget that the primary function of the skeleton is locomotion of the body and that only adequately rigid and strong bones make this vital function feasible [16]. It is thus important to evaluate the effects of CsA on bone mechanical properties. Warren et al. treated the rats with CsA for 14 consecutive days at a dose of 7 mg/kg body weight/day, and did not find alteration in biomechanical properties with respect to the failure torque and the stiffness of the femur diaphysis [17]. However, they reminded us that testing in torsion emphasizes cortical bone characteristics, and the short-term nature of treatment with this drug may have failed to significantly influence cortical bone [17]. It is still necessary to illuminate the detrimental effect of CsA on bone mechanical proprieties in rats.

In the present study, we hypothesized that the long-term use of CsA in the rats will damage the bone mechanical properties, and the detrimental effect of CsA on biomechanical properties is different in varied skeletal anatomy sites.

## Methods

### Animals

Fifty-six male 3-month-old Wistar rats (200~250 g) purchased from the Model Animal Research Center of Nanjing University were housed with free access to water and chow with constant 12-hour light-dark cycle environmental conditions. They were all acclimatized for 1 week before the experiment began. All animal experiments were approved by the Animal Study Committee of Nanjing University.

Eight rats were randomly chosen as the basal group and were sacrificed at the beginning of the injection protocol for the treated rats, while the others were randomly distributed into four groups of 12 animals each. One group was used as controls and received daily subcutaneous injection of 1 ml of saline solution. Another three experimental groups were injected subcutaneously with CsA (Novartis Pharmaceuticals, Basel, Switzerland) in a daily dose of 1.5 mg/kg body weight, 7.5 mg/kg body weight, and 15 mg/kg body weight respectively [8,11,18]. The experimental period of the study extended over 60 days.

### Biochemical assay of blood and body weight measurement

After sixty days of the CsA therapy, the rats were exsanguinated from the abdominal aorta under anesthesia to obtain serum for the determination of serum alanine aminotransferase (ALT), serum aspartate aminotransferase (AST), serum urea nitrogen (BUN), and serum creatinine (Cr). The body weight was also measured [19].

### Evaluation of kidney and liver histologic changes

For histological analysis of the rat's kidney and liver in 15 mg/kg CsA group, paraffin-embedded 6- μm-thick sections were stained with hematoxylin and eosin [19].

### Bone mineral density and bone length measurement

**Figure 1:**
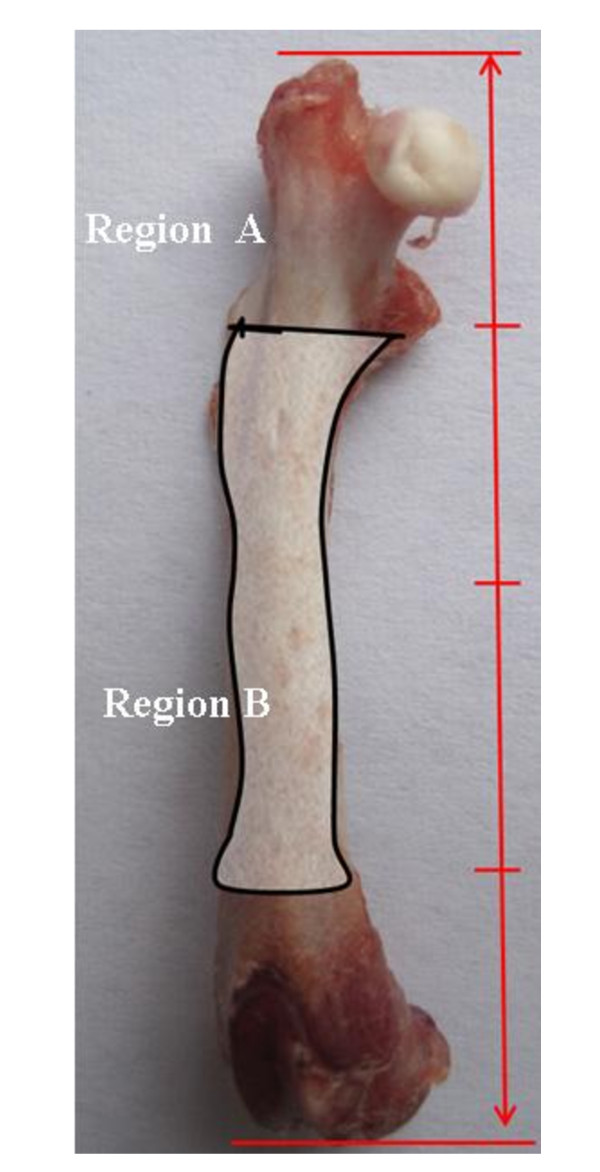
**The region defined on the femur**. Region A: the proximal femur region, the proximal one of four equal regions of the femur length; Region B: the femur mid-diaphseal region, the middle two of the four equal regions of the femur length.

As we reported in our previous study [19], the L_1-5 _vertebra and the total femur were removed off the soft tissue, and the bone mineral density (BMD) of the L_1-5 _vertebra, the femur mid-diaphseal region (the middle two of the four equal regions of the femur length, Figure 1) and the proximal femur region (the proximal one of four equal regions of the femur length, Figure 1) were measured using dual-energy X-ray absorptiometry (Hologic QDR4000, USA). The lengths of humerus, tibia and femur were measured using vernier caliper.

### Mechanical testing

The mechanical test was described in detail in our previous study [19]: the extrinsic parameters (the ultimate force, the stiffness and the energy absorption) were obtained directly from the load-deformation curves that were recorded continually in a computerized monitor and the intrinsic parameters (the ultimate stress, the Young's modulus and the energy absorption per unit of bone volume) can be computed from extrinsic parameters and geometric data. Briefly, a compression force was applied to the cylinder samples of the L_2 _body specimen at a rate of 2.5 mm/minute (Instron 3367); the L_2 _body cross sections were assumed to be elliptically shaped, two diameters, anterior-posterior (a) and left-right (b), of this cylinder were measured using vernier caliper and used to calculate the cross-sectional area (S) (S = πab/4). A bending force was applied to the middle femur shaft at a speed of 5 mm/minute until fracture occurred; the femur cross sections were assumed to be elliptically shaped, the moment of inertia is I = π[ab^3^-\(a-\2t)(b-\2t)^3^]/64: Where a is the width of the cross section in the medial-lateral direction, b is the width in the anterior-posterior direction, and t is the average cortical thickness. A cantilever bending force was applied perpendicularly to the femur neck at a speed of 5 mm/minute until fracture occurred; the femur neck cross sections were assumed to be elliptically shaped, the section modulus is Wz = πa^2^b/32: Where a is the width of the cross section in the superior-inferior direction, and b is the width in the anterior-posterior direction.

### Bone histomorphometry analyses

The L_4 _vertebra body and the right proximal tibia metaphysis were removed off the soft tissue and fixed in 10% phosphate-buffered formalin for 24 h. The vertebrae and the tibia metaphyses were then dehydrated in ethanol, defatted in xylene, and embedded in methyl methacrylate. The frontal sections were cut at 4 μm thickness with a microtome (Leica RM2155; Germany) and were stained with Goldner's trichrome for quantification of trabecular bone architecture. A semi-automatic image analysis system (Image-Pro Plus) was used to measure the bone volume fraction(%), the trabecular number (#/mm), the trabecular thickness (μm), and the trabecular separation (μm) [20]. The anterior-posterior cortical thickness of the middle femoral shaft was measured using vernier caliper [19].

### Statistical analysis

All statistical analyses were carried out using SPSS v.13. The groups were compared by means of 1-way ANOVA (S-N-K) if the variances were homogeneous; but if not, Kruskal-Wallis test was used. The statistical differences were detected among the control group, 1.5 mg/kg CsA group and 7.5 mg/kg CsA group. All values were expressed as $\bar{X}\pm$ SD. Statistical significance was accepted at P < 0.05.

## Results

### Effects of varying doses of CsA on kidney and liver function

**Figure 2:**
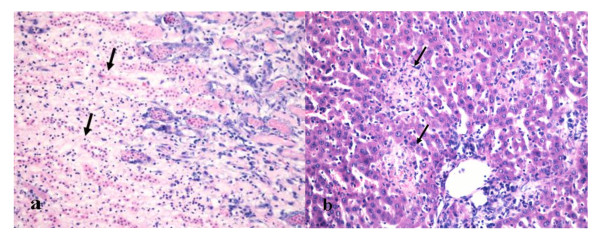
**Histological examinations of the kidney and liver**. Only one rat survived in the 15 mg/kg CsA group. The kidney and liver histology of the survived rat showed tissue necrosis: (a) Prominent tubular necrosis (black arrow) was found in the renal section; (b) Local necrosis (black arrow) was detected in the liver section (HE, Original magnification: ×200).

In the 15 mg/kg CsA group only one rat survived. The kidney and liver histology of the survived rat showed extensive tissue necrosis (Figure 2), and the serum ALT, AST, BUN and Cr levels were respectively 2.5, 2.8, 3, and 1.5 times of the averaged levels of the control group. In the 7.5 mg/kg CsA group, three rats died. One rat in the 1.5 mg/kg CsA group was excluded in our study due to a huge neck mass. No significant change in ALT, AST, BUN and Cr levels was observed among the control group, the 1.5 mg/kg CsA group and the 7.5 mg/kg CsA group (Table 1).

**Table 1:** Effects of CsA on the liver and kidney function

	ALT (U/L)	AST (U/L)	BUN (mmol/L)	Cr (umol/L)
Baseline(n = 8)	47.4 ± 6.2	174.8 ± 34.7	8.0 ± 2.1	79.4 ± 12.3
Control(n = 12)	50.0 ± 6.2	191.3 ± 51.3	8.4 ± 1.8	82.3 ± 10.7
1.5 mg CsA(n = 11)	45.3 ± 8.3	158.6 ± 82.9	9.3 ± 1.5	75.8 ± 15.7
7.5 mg CsA(n = 9)	72.5 ± 26.3	211.2 ± 121.3	11.8 ± 5.0	86.3 ± 17.5

Data are expressed as means ± SD.

### The kidney and liver histology of the survived rat in 15 mg/kg CsA group

Only one rat survived in the 15 mg/kg CsA group. The kidney and liver histology of the survived rat showed tissue necrosis (Figure 2).

### Effects of varying doses of CsA on the bone length and the body weight

**Figure 3:**
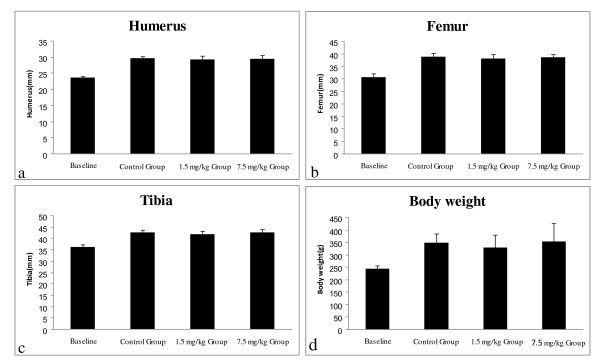
**Effects of CsA on the bone length and the body weight**. No significant change in the bone length or the body weight was observed among the control group, the 1.5 mg/kg CsA group and the 7.5 mg/kg CsA group: (a) The bone length of humerus; (b) The bone length of femur; (c) The bone length of tibia; (d) The body weight. Data are expressed as means (bars) ± SD (error bar).

No significant change in the bone length or the body weight was observed among the control group, the 1.5 mg/kg CsA group and the 7.5 mg/kg CsA group (Figure 3).

### Effects of varying doses of CsA on bone mineral density

Compared with the control group, both the 1.5 mg/kg CsA group and 7.5 mg/kg CsA groups had statistically significant decreases in the BMD and BMC values of the lumber vertebra and the proximal femur, but had no significant changes in the BMD and BMC values of the middle femur. No significant differences in the values of the Area were obtained among the control group, 1.5 mg/kg CsA group and 7.5 mg/kg CsA group (Table 2).

**Table 2:** Effect of CsA on the bone mineral density.

	BMD (g/cm^2)^	BMC (g)	Area (cm^2)^
Lumbar Vertebra			
Baseline (n = 8)	0.237 ± 0.018	0.502 ± 0.073	2.124 ± 0.287
Control (n = 12)	0.259 ± 0.026	0.609 ± 0.107	2.343 ± 0.297
1.5 mg CsA (n = 11)	0.204 ± 0.030**	0.473 ± 0.091**	2.325 ± 0.260
7.5 mg CsA (n = 9)	0.201 ± 0.015**	0.462 ± 0.073 **	2.291 ± 0.462
Proximal Femur			
Baseline (n = 8)	0.245 ± 0.038	0.126 ± 0.022	0.513 ± 0.028
Control (n = 12)	0.258 ± 0.046	0.139 ± 0.029	0.536 ± 0.029
1.5 mg CsA (n = 11)	0.181 ± 0.036*	0.099 ± 0.017**	0.542 ± 0.037
7.5 mg CsA (n = 9)	0.171 ± 0.039**	0.092 ± 0.025**	0.530 ± 0.027
Middle Femur			
Baseline (n = 8)	0.298 ± 0.075	0.228 ± 0.058	0.762 ± 0.021
Control (n = 12)	0.323 ± 0.068	0.258 ± 0.052	0.794 ± 0.058
1.5 mg CsA (n = 11)	0.311 ± 0.066	0.241 ± 0.058	0.771 ± 0.052
7.5 mg CsA (n = 9)	0.317 ± 0.058	0.243 ± 0.045	0.767 ± 0.065

Data are expressed as means ± SD. Compared with the control group, p < 0.05 *, p < 0.01 **.

### Effects of varying doses of CsA on bone mechanical properties

The second lumbar vertebra displayed statistically significant lower values of the ultimate force, the ultimate stress and the energy absorption per unit of bone volume between the control group and the two experimental groups treated with 1.5 mg/kg and 7.5 mg/kg CsA respectively (Table 3).

**Table 3:** Effects of CsA on the mechanical parameters of the lumbar vertebra

	Baseline (n = 8)	Control (n = 12)	1.5 mg CsA (n = 11)	7.5 mg CsA (n = 9)
Ultimate force (N)	143.7 ± 24.4	156.9 ± 36.2	119.9 ± 34.7*	113.6 ± 45.8*
Stiffness (N/mm)	688.2 ± 209.7	756.1 ± 258.6	698.7 ± 298.3	547.4 ± 250.1
Energy absorption (mJ)	29.8 ± 14.5	36.4 ± 16.9	24.8 ± 11.7	23.9 ± 13.8
Ultimate stress (GPa)	0.017 ± 0.003	0.017 ± 0.004	0.013 ± 0.003*	0.011 ± 0.005**
Young's modulus (Gpa)	0.488 ± 0.160	0.444 ± 0.130	0.454 ± 0.213	0.333 ± 0.147
Energy absorption (mJ/mm3)	0.81 ± 0.34	0.77 ± 0.44	0.44 ± 0.24*	0.34 ± 0.28*

Data are expressed as means ± SD. Compared with the control group, p < 0.05 *, p < 0.01 **.

Compared with the control group, the middle femur shaft of the experimental group treated with 1.5 mg/kg or 7.5 mg/kg CsA showed no significant change in the value of any of the index of the bone biomechanical proprieties (Table 4).

**Table 4:** Effects of CsA on the mechanical parameters of the middle femurs

	Baseline (n = 8)	Control (n = 12)	1.5 mg CsA (n = 11)	7.5 mg CsA (n = 9)
Ultimate force (N)	112.7 ± 19.1	139.9 ± 23.1	128.6 ± 22.1	131.8 ± 20.3
Stiffness (N/mm)	317.6 ± 87.4	423.6 ± 95.3	347.5 ± 96.4	396.6 ± 89.8
Energy absorption (mJ)	20.1 ± 4.5	23.6 ± 5.4	24.7 ± 5.4	22.9 ± 4.8
Ultimate stress (GPa)	0.098 ± 0.020	0.101 ± 0.016	0.101 ± 0.013	0.096 ± 0.016
Young's modulus (Gpa)	9.9 ± 2.5	10.9 ± 2.5	9.2 ± 1.5	9.6 ± 2.3
Energy absorption (mJ/mm3)	0.49 ± 0.08	0.46 ± 0.07	0.53 ± 0.14	0.47 ± 0.08

Data are expressed as means ± SD.

Compared with the control group, the femoral neck of rats treated with 7.5 mg/kg CsA showed significantly lower values of the ultimate force, the ultimate stress and the Young modulus. However, the femoral neck of rats treated with 1.5 mg/kg CsA only showed a definite downward trend in all indices of the bone biomechanical proprieties, but the difference did not reach the statistical significance (Table 5).

**Table 5:** Effects of CsA on the mechanical parameters of the femoral neck

	Baseline (n = 8)	Control (n = 12)	1.5 mg CsA (n = 11)	7.5 mg CsA (n = 9)
Ultimate force (N)	59.3 ± 11.1	70.4 ± 7.2	62.9 ± 13.2	54.9 ± 14.8*
Stiffness (N/mm)	108.4 ± 23.1	115.0 ± 21.3	103.3 ± 20.2	105.6 ± 26.8
Energy absorption (mJ)	31.0 ± 10.4	30.3 ± 7.0	28.8 ± 11.1	27.9 ± 15.5
Ultimate stress (GPa)	0.178 ± 0.016	0.196 ± 0.010	0.175 ± 0.033	0.135 ± 0.026**
Young's modulus (Gpa)	2.25 ± 0.47	2.37 ± 0.39	2.08 ± 0.54	1.67 ± 0.55**

Data are expressed as means ± SD. Compared with the control group, p < 0.05 *, p < 0.01 **.

### Effects of varying doses of CsA on the trabecular bone architecture

**Figure 4:**
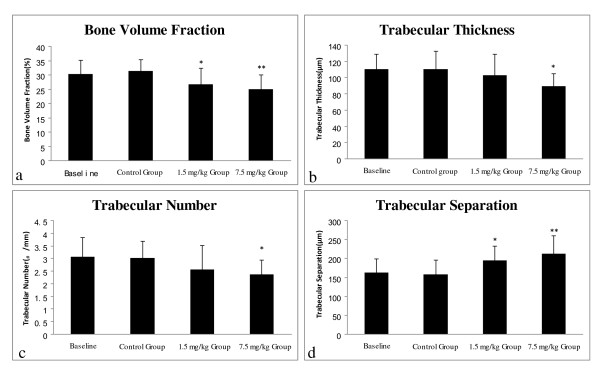
**Effects of CsA on the lumbar vertebral trabecular bone architecture**. Compared with the control group: (a) The values of the bone volume fraction were significantly decreased in the 1.5 mg/kg and the 7.5 mg/kg CsA group; (b) The values of the trabecular thickness were significantly decreased in the 7.5 mg/kg CsA group; (c) The values of the trabecular number were significantly decreased in the 7.5 mg/kg CsA group; (d) The values of the trabecular separation were significantly increased in the 1.5 mg/kg and the 7.5 mg/kg CsA group. Data are expressed as means (bars) ± SD (error bar). Compared with the control group: p < 0.05 *, p < 0.01 **.

The fourth lumbar vertebra displayed statistically significant lower values of the bone volume fraction, the trabecular thickness and the trabecular number between the control group and the 7.5 mg/kg CsA group. However, the fourth lumbar vertebra of the rats of the 1.5 mg/kg CsA group only showed statistically significant lower values of the bone volume fraction. Compared with the control group, the fourth lumbar vertebra of both experimental groups showed significantly higher values of the trabecular separation (Figure 4).

**Figure 5:**
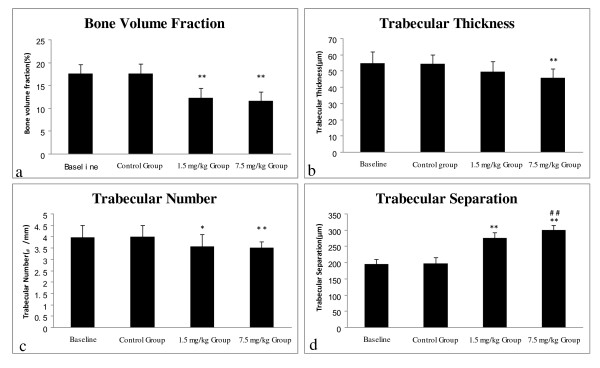
**Effects of CsA on the proximal tibia metaphysis trabecular bone architecture**. Compared with the control group: (a) The values of the bone volume fraction were significantly decreased in the 1.5 mg/kg and the 7.5 mg/kg CsA group; (b) The values of the trabecular thickness were significantly decreased in the 7.5 mg/kg group; (c) The values of the trabecular number were significantly decreased in the 1.5 mg/kg and the 7.5 mg/kg CsA group; (d) The values of the trabecular separation were significantly increased in the 1.5 mg/kg and the 7.5 mg/kg CsA group; Compared with the 1.5 mg/kg CsA group, the values of the trabecular separation were also significantly increased in the 7.5 mg/kg CsA group. Data are expressed as means (bars) ± SD (error bar). Compared with the control group: p < 0.05 *, p < 0.01 **. Compared with the 1.5 mg/kg CsA group: p < 0.05#, p < 0.01##.

The right proximal tibia metaphysis displayed statistically significant lower values of the bone volume fraction, the trabecular thickness and the trabecular number between the control group and the 7.5 mg/kg CsA group. However, the right proximal tibia metaphysis of the rats of the 1.5 mg/kg CsA group only showed statistically significant lower values of the bone volume fraction and the trabecular number. Compared with the control group, the right proximal tibia metaphysis of both experimental groups showed significantly higher values of the trabecular separation (Figure 5).

### Effects of varying doses of CsA on the cortical thickness of the femoral shaft

**Figure 6:**
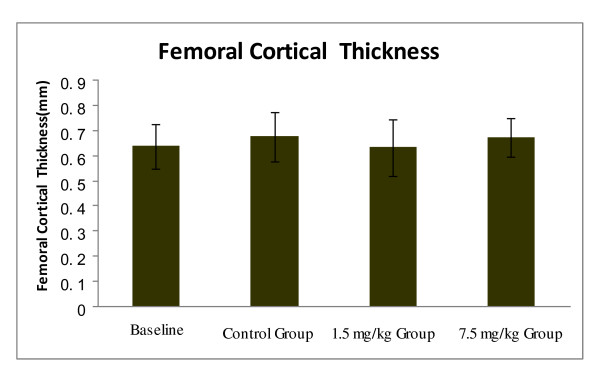
**Effects of CsA on the femoral cortical thickness**. No significant change in the femoral cortical thickness was observed among the control group, the 1.5 mg/kg CsA group and the 7.5 mg/kg CsA group. Data are expressed as means (bars) ± SD (error bar).

No significant change was observed in the cortical thickness of the middle femoral shaft between the control group and the two experimental groups (Figure 6).

## Discussion

The patients who have undergone organ transplantation or acquired autoimmune disease always suffer from bone loss [21-24]. It is thus important to find out whether the long-term use of CsA will increase these patients' fracture risk. The present study showed that the CsA therapy at 7.5 mg/kg significantly reduced the BMD, the ultimate force and the ultimate stress of the lumbar vertebra and the proximal femur. The results indicated that the CsA therapy at 7.5 mg/kg can damage the mechanical properties and thus may increase the fracture risk of the lumbar vertebra and the proximal femur. Such findings were consistent with those reported by Epstein et al [25], and may have important clinical implications.

Osteoporotic fracture is widely recognized as a public health threat because it is associated with increased risk of morbidity and mortality, and with significant health care costs [26,27]. Even in normal population, vertebral fractures and hip fracture are substantially associated with the mortality risk. Some recent studies reported that patients with osteoporotic vertebral fractures have a significant four-fold increase in mortality compared with patients without vertebral fractures [28], and that the mortality after a hip fracture remains significantly higher, being 11-23% at six months and 22- 29% at one year from injury [29]. The present results also supported that if the CsA therapy is applied, the change of BMD of the vertebra and the hip should be monitored to predict the fracture risk.

The effects of CsA on the rat skeleton biomechanical properties closely bear upon both the dose and the duration of administration [10,11]. Goodman et al. found that the rats receiving 7.5 mg/kg CsA by daily oral gavages for 84 days showed osteopenia, and the rats receiving 1.5 mg/kg CsA were relatively bone sparing [8]. Our results showed that the CsA therapy at either 1.5 mg/kg or 7.5 mg/kg would damage the biomechanical properties of the lumbar vertebra, but neither dose of CsA therapy had a detrimental effect on the middle femur, furthermore, CsA therapy at 7.5 mg/kg but not at 1.5 mg/kg could damage the biomechanical properties of the proximal femur. Our findings importantly suggested that apart from the doses and duration of CsA therapy, the effects of CsA on skeletons were also closely related to the selection of anatomic sites, that is, the effects of CsA on skeleton are site-specific.

It is well known that the structural and metabolic heterogeneity is salient in the bones of rats. The cancellous bone is mainly located in axial bones with high turnover, while the cortical bone is mainly found in long bones with low turnover. Our results showed that compared with the control group, in the 7.5 mg/kg CsA therapy group the values of the bone volume fraction, the trabecular thickness, and the trabecular number of the lumbar vertebra were significantly reduced, whereas the value of the middle femur cortical thickness had no statistically significant change. The variations of these trabecular bone architectures were consistent with the changes of the BMD and biomechanical proprieties in the 7.5 mg/kg CsA therapy group. This is so because compared with the control group, the values of the BMD, the ultimate force, the ultimate stress and the energy absorption per unit of bone volume of the lumbar vertebra in this therapy group were significantly reduced, whereas these values of the middle femur had no significant change. Our findings suggested that the lumbar vertebra contained predominantly cancellous bone and was prone to damage by the CsA, while the middle femur contained mainly cortical bone and was resistant to the deterioration of CsA. Our findings are in accordance with Movsowitz et al.'s report that CsA in the dose of 7.5 mg/kg could induce high turnover osteoporosis in rats, and the bone loss mainly occurred in cancellous bone [11]. Our findings are also in agreement with Klein et al.'s findings that CsA has little effect on cortical bone in rats [9].

CsA therapy is also associated with renal and hepatic impairment. In the 15 mg/kg CsA group only one rat survived. The kidney and liver histology of the survived rat showed extensive tissue necrosis, and the serum ALT, AST, BUN and Cr levels were respectively 2.5, 2.8, 3, and 1.5 times of the averaged levels of the control group. The eleven rats may have died from the renal and hepatic impairment. In the 7.5 mg/kg CsA group, three rats died. Although we could not ascertain the exact cause for their death, these rats manifested the same cachexia characteristics before their death as the rats that died in the 15 mg/kg CsA group. We thus speculated that these rats may have also died from the renal and hepatic damage. Compared with the control group, no significant change in body weight and bone length was observed in either the 1.5 mg/kg or the 7.5 mg/kg CsA therapy group. This may suggest that CsA therapy at 1.5 mg/kg or 7.5 mg/kg has no inhibitory effect on the rat body and bone growth.

The exact mechanism that leads to the deterioration of skeletal biomechanical properties after CsA therapy remains inconclusive. In-vivo studies have demonstrated that the parameters of osteoblast and osteoclast function are both increased [10,30], and our results have shown that CsA therapy at 1.5 and 7.5 mg/kg has no inhibitory effect on rat bone growth. Thus, it is unlikely that CsA has had a direct toxic effect on bone cells. In all likelihood, it maybe a combination of the two possibilities: 1) CsA may act directly on bone cells and affect their ability to secrete local autocrine factors or respond to systemic hormones. 2) CsA may have an indirect action on bone via their effects on the immune system [30,31]. Considering that the kidney is closely related to the bone mineral metabolism [32], we speculated that CsA therapy, especially in high dose, may have an indirect action on bone through a mechanism of abnormal mineral homeostasis due to impaired kidney function.

It has been reported that bone growth in rats slows drastically at 3 months of age and plateaus by 12 months [33], and that 3-month-old rats are more sensitive to the CsA therapy, apart from being in much better health condition and thus able to endure long-term CsA therapy [34,35]. In light of the previous reports, we used 3-month-old rat as the animal model in our present study. This animal model shares many characteristics with its human analog of post-transplantation osteoporosis, including a similar pattern of bone loss and a positive response to bisphosphonates therapy that is beneficial to humans [7]. However, the characteristics of bone remodeling in cortical bone in rats are not congruent to the human case [33]. The lack of intracortical bone remodeling in cortical bone may contribute to the resistance of the femur diaphysis to the damage of CsA therapy. Therefore this model should not be used to assess the response of cortical bone to the CsA therapy.

## Conclusions

Long-term use of CsA can weaken the biomechanical properties and thus increase the fracture rate of the lumbar vertebra and the proximal femur. However, CsA therapy has less effect on the middle femur shaft. The effects of CsA on skeleton are site-specific.

## Competing interests

The authors declare that they have no competing interests.

## Authors' contributions

YXC designed the study and drafted the manuscript. XZ, RZ and JFW performed the experimental work and the statistical analysis. All authors have read and approved the final manuscript.

## Pre-publication history

The pre-publication history for this paper can be accessed here:

http://www.biomedcentral.com/1471-2474/12/240/prepub
